# Social determinants of rest deprivation amongst Ghanaian women: national and urban-rural comparisons with data from a cross-sectional nationally representative survey

**DOI:** 10.1186/1471-2458-10-580

**Published:** 2010-09-28

**Authors:** Maurice B Mittelmark, Torill Bull

**Affiliations:** 1 Department of Health Promotion and Development, Faculty of Psychology, University of Bergen, Christiesgt. 13, 5020 Bergen, Norway

## Abstract

**Background:**

Rest deprivation (rest/napping/sleep 6 or less hours daily) is a clinically recognised risk factor for poor health, but its epidemiology is little studied. This study reports prevalence's and social correlates of rest deprivation in Ghana.

**Methods:**

Data are from the 2008 Ghana Demographic and Health Survey. Women ages 15-49 were recruited in a national sampling design. Respondents were 4,916 women in the national sample, a sub-sample of 530 women in the three northernmost rural regions and a sub-sample of 853 women in urban Greater Accra.

**Results:**

Prevalence's of rest deprivation were 0.13% nationally, 14.5% in Greater Accra and 16.8% in the North. The significant correlates nationally were age, education, wealth index, Christian religion and literacy. In Accra, they were age, wealth index, having household electricity, and possession of a refrigerator, a stove and a mobile phone. In the North, they were education, occupation, drinking water source, possession of motorcycle/scooter, Christian religion, literacy, and possession of a clock and a cupboard. In logistic regression analyses controlling for age in the national sample, the significant odds ratios were 1.40 for no education compared to secondary and higher education, 0.78-0.43 for the four poorer wealth quintiles compared to the richest wealth index quintile, and 0.55 for Christian religion compared to all others.

Also controlling for age, the significant odds ratios in Accra were 2.15 for the second richest wealth quintile compared to the richest quintile and 0.16 for possession of a mobile phone. In the North they were 0.49 for Christian religion compared to all others, 1.87 for having a protected compared to an unprotected water source, and 0.41 for having a cupboard in the home.

**Conclusions:**

Education, wealth and religion were related to rest deprivation nationally but not in the urban and rural regions (except for religion in the North). This suggests caution in generalising about the social correlates of rest deprivation at a regional level, based on national-level data. Qualitative research in local contexts is needed in order to illuminate the social determinants of rest pattern, and to provide guidance about better ways to measure such determinants in future survey research.

## Background

The terms short sleep duration, sleep insufficiency and sleep restriction are synonymous concepts described in the medical and public health literatures, all referring to inadequate quantities of daily, contiguous sleep. Many studies show that the optimal sleep duration is 7-8 hours [1-3], with negative mental and physical health effects at sleep durations both below and above this optimal level [1,2,4-10]. There is a long line of evidence from laboratory research that adequate sleep is essential to good immune functioning [11,12], and this may help explain the wide range of health problems that are associated with poor sleep. However, the evidence on the association of poor sleep and poor health and functioning is not entirely consistent. For example, subjective well-being has been observed both to vary [13,14] and not to vary with sleep duration [15], and the same is true for mortality [16,17].

Compared to the clinical evidence on sleep and health, the epidemiology of sleep and health is relatively unexplored. Few population-based studies provide prevalence estimates and assess putative risk factors for sleep insufficiency. Of those that are in the literature, one by Soldatos [18] provides international comparisons. In data from 11 countries, the highest rate of sleep insufficiency was reported in Belgium (23.2%) and the lowest rate was in Austria (10.4%), indicating a high level of heterogeneity. Athens Insomnia Scale [18] ratings showed significant age differences with older respondents reporting higher levels of night awakenings, waking earlier than desired, less total sleep duration and less sleepiness during the day [19]. Another international study observed that higher wages were associated with lower sleep time for men but not for women and the (non-significant) trend for women was the reverse of that for men [20].

At the national level, in 2002 data from the USA's Behavioral Risk Factor Surveillance System (BRSS), 26% of adults reported sleep insufficiency during 14 or more of the past 30 days [14]. In the same study, the prevalence of sleep insufficiency was significantly lower with age, higher for women, slightly higher with higher education, and higher for never married respondents and for those not able to work. The findings for age seem at odds with the findings of Soldatos, et al., [18], but that is not necessarily so, since the Soldatos, et. al. [18] study queried respondents about sleep patterns but not about self-perceived sleep sufficiency. Older people may experience less contiguous sleep than younger people experience, yet judge that their sleep is not problematic. In the 2002 BRSS, sleep insufficiency was significantly associated with poor self-reported health, physical and mental distress, depression and anxiety symptoms, pain and activity limitations.

In USA data from the 2001 Sleep in America Poll's nationally representative sample, significant correlations ranging from 0.24 to 0.51 were observed among five sleep problems: difficulty falling asleep, waking too early, daytime sleepiness, waking during the night and awaking unrefreshed [3]. In a nationally representative study in Britain in 1997, 58% reported sleep problems one or more nights the previous week and 18% reported sleep insufficiency the majority of nights [13]. In the same study, older people reported sleeping less, women reported more sleep problems, and quality of life was diminished for those sleeping short and long hours.

The most recent large-scale survey of sleep is from the 2006 BRSS [21]. Importantly, this was the first public health research project to recognise the importance to health and functioning of other forms of rest than nighttime sleeping. In many parts of the world, daily naps are an important component of restorative activity, as is the ability to take sufficient pauses from work. Regarding napping, one international study observed an overall napping prevalence of 23.1% and prevalence ranged from a high of 42.4% in Brazil to a low of 12% in Japan [19]. In the 2006 BRSS, respondents were asked "During the past 30 days, for about how many days have you felt you did not get enough rest or sleep?" In the combined data from four US states, insufficient rest or sleep during the previous 14 days was reported by 24.9% of respondents [21]. In the same data, the prevalence of rest/sleep problems was lower with age, higher for women, highest amongst those unable to work and higher amongst those with higher education levels.

### Living conditions

While education and employment have been examined for associations with sleep insufficiency in some studies, very few have also examined income/expenditure/wealth, which is the *de rigueur *indicator of social living conditions. The importance of living conditions to health has received greatly increased attention recently, with the issuance in 2008 of the World Health Organisation Commission on the Social Determinants of Health report "Closing the Gap in a Generation: Health Equity Through Action on the Social Determinants of Health [22]. The report emphasises the health inequities experienced by women, and amongst its major recommendations the report calls for civil society, governments, and global institutions to "Improve the well-being of girls and women and the circumstances in which their children are born, [...] improve living and working conditions and create social protection policy supportive for all..."

The only study reviewed above to examine the relationship of sleep insufficiency to income as well as education was an international economics study in which higher wages were associated with lower sleep time for men but not for women and the (non-significant) trend for women was the reverse of that for men [20]. The Alameda County Health and Ways of Living Study (California, USA) also examined the relationship of living conditions indicators and sleep insufficiency [9]. The age-adjusted odds of short sleep duration was 1.62 (CI 1.34-1.94) for respondents in the lowest household income quintile compared to the highest quintile, and the odds of short sleep duration were 1.51 for respondents with less than a high school education, compared to better educated respondents. We are aware of no other community-based study that has examined the association of sleep quantity with living conditions. Only the 2006 BRSS has examined rest and sleep sufficiency, but that study did not include a measure of income [21].

The studies referred to above are part of a very large literature extending back more than four decades, showing that too little rest is a risk factor for poor health. However, there are some gaps in the literature, including four that this paper focuses on.

The first is that the known social determinants of health generally - income, education and occupation amongst other indicators of living conditions - have hardly been examined in sleep research. A second gap is the paucity of data from the Global South. Most sleep studies are from a few industrialised countries that enjoy the highest socioeconomic status in the world, findings from which may generalise poorly to the Global South [23]. Third, sleep is but one aspect of regenerative health; rest and napping in combination with adequate sleep (and adequate physical activity, water intake and diet) are essential to healthy functioning. However almost nothing is known about patterns of rest including sleeping, but also relaxing and napping. Finally, national-level public sleep health studies may mask important regional differences, and regional-level analyses may be required to inform local policy and practice. A large degree of international variation was described above, and the 2006 BRSS suggests the same for regional variation. In those data from four US states, insufficient rest or sleep 14 or more days out of 30 was reported by a low of 20.8% of respondents in Delaware and a high of 27.4% of respondents in Rhode Island, two states that are not otherwise conspicuously different [21]. If prevalence rates vary regionally, and if living conditions are associated with sleep sufficiency, then varying living conditions in regions may explain part of the variation in sleep sufficiency. This is an empirical issue, which can only be approached with regional-level analyses.

This study aimed to investigate the prevalence of rest deprivation (6 and less hours daily of combined rest, napping and sleep) amongst Ghanaian women nationally and in two regions, the urban capital area of Greater Accra and the mostly rural regions Upper East, Upper West and the Northern region (referred to collectively hereafter as North). We investigated also the degree to which the living conditions of the women were related to rest deprivation. Living conditions were conceptualised and measured in the standard way for research in developing countries; the key indicators are ownership of material goods and housing quality (wealth index) and levels of education and occupation. Wealth replaces income, which is used in research in the Global North, since livelihoods in the Global South are typically a mix of activities, only some of which may generate income. Barter, trade and labour exchange are important elements in livelihood generation in developing countries. In addition to the three key living conditions indicators, the relationship of rest deprivation to a wide range of other living conditions indicators was examined in this exploratory study, including religion, literacy and land and livestock ownership.

## Methods

This article reports analyses using data from the 2008 Ghana Demographic and Health Survey (GDHS) [24]. The survey collected data on housing and household characteristics, education, maternal health and child health, nutrition, family planning, gender, and knowledge and behaviour related to HIV/AIDS, living conditions, and combined hours of daily rest, napping and sleep. The 2008 GDHS was conducted by the Statistical Service of Ghana and the Ministry of Health/Ghana Health Service. Required ethical clearances were obtained by the Statistical Service of Ghana from the Ghana Health Service Ethical Review Committee in Accra, Ghana. Informed consent for the survey was obtained from the respondent at the beginning of the data collection interviews.

### Context

Even if the last decades have brought some economic development to Ghana, the country is still one of the poorest in the world. In 2009, it ranked as number 152 of 182 countries on the Human Development Index [25]. Fifty-four percent of the population live in rural areas, the life expectancy is 57 years at birth, there is a high under-five mortality (112 per 1000), and a high maternal mortality (214 per 100 000 live births [26]. Ghana is divided into ten political regions. An important characteristic of Ghana is the strong divide when it comes to level of development between the lush south of the country and the poorer, arid and less developed north. Administration and business is located in the south.

### Study design

The 2008 GDHS had a two-stage stratified sampling design [24]. The sample units were 412 geographical clusters producing a national and regional representative sample with proportional representation of rural and urban populations. At the second stage of sampling, households were selected within each cluster after a complete household enumeration. The objective was the achievement of representative samples at urban-rural, regional and national levels, large enough to allow for precise estimates of key indicators. To adjust for non-responses at all levels and for the stratified sampling design, sample weights were calculated. 12,360 households were selected. In half of these households all women aged 15-49 were eligible for interview, and one woman in each household was selected at random and interviewed by a trained interviewer. The national sample of women for the current study was 4,916. However, 65 of these were not *de jure *residents of the households where they were interviewed. Respondents who were *de facto *but not *de jure *residents are not included in the present study. Also studied were the sub-sample of all 530 respondents in the three northernmost rural regions and the sub-sample of all 853 respondents in urban Greater Accra.

### Study variables

For the *rest deprivation *variable the respondents were asked 'How many hours do you rest a day, including naps and sleep both during the day and night (response frame 1-3 hours, 4-6 hours, 7-9 hours, 10 and more hours, don't know)? For this study, this variable was dichotomized into ≤ 6 hours (rest deprivation) and ≥ 7 hours (rest sufficiency). Hereafter the terms 'rest deprivation' and 'rest sufficiency' are used as labels for the two values of the binomial rest variable. For *age*, a variable with five-year categories was used. For *wealth *the DHS Wealth Index was used, which is constructed using a principle components analysis to score a household's quality and assets. For the GDHS 2008 these assets included ownership of consumer items such as television, bicycle, car, and also dwelling characteristics such as drinking water, sanitation facilities, and flooring material. Each household was given a wealth score, and this score was assigned to each individual within the household. The national sample was divided into quintiles from the richest to the poorest, and these quintiles were used in this study. The Wealth Index was recalculated for each sub-sample. *Education *was measured by a three-category variable (secondary or higher, primary, no education). To create the *occupation *variable, occupational categories from GDHS were dichotomized into 'white collar' (professional/technical/managerial, clerical, sales and services) and 'agriculture/labour' (agriculture self-employed, agriculture, skilled manual labour and unskilled manual labour, household/domestic labour).

In addition to education, occupation and wealth, other putative living conditions indicators were examined in this study. All of the assets that composed the Wealth Index were also separately evaluated for their relationship to the rest variable. The living conditions indicators included also land ownership and livestock ownership (both coded yes/no). For religion, the GDHS variable was recoded into a dichotomy of 'Christian' (Catholic, Anglican, Methodist, Pentecostal/Charismatic, other Christian) and 'Moslem/other' (Moslem, Traditionalist/other, no religion). During interviews, literacy was tested with a reading card. Three categories were recoded into a dichotomy of 'can read' (able to read only parts of sentence, able to read whole sentence) or 'cannot read' (cannot read at all).

The data were weighted in this way: "There are two main sampling weights in DHS surveys: household weights and individual weights. The household weight for a particular household is the inverse of its household selection probability multiplied by the inverse of the household response rate of its household response rate group. The individual weight of a respondent's case is the household weight multiplied by the inverse of the individual response rate of her individual response rate group." [[27], page 12]

SPSS 15 was used for all analyses. The Chi-square test was used to test associations between rest deprivation and all the living conditions indicators. Binary logistic regression was used to compute odds ratios and 95% confidence intervals, separately for all three samples, in analyses including all the living conditions indicators having a significant two-way relationship to the rest variable.

## Results

### Respondents

The 4,916 women survey respondents were distributed by age as follows: 15-19 (20.8%); 20-24 (17.9%); 25-29 (16.9%); 30-34 (13.1%); 35-39 (13.0%); 40-44 (9.6%); 45-49 (8.7%). The sample sizes from the ten regions ranged from a low of 334 in the Central region to a high of 815 in the Ashanti region. The weighted sample size ranged from 122 in the Upper West region to 1,011 in the Ashanti region. Sixty-five respondents were not resident in the households they were interviewed in and were excluded from all analyses. Excluding these *de facto *residents, missing data rates ranged from a low of 0.0% to a high of 1.7% for the variables included in this study. All analyses reported from this point forward were conducted with weighted data.

Prevalence's of rest deprivation were 13.0% nationally, 14.5% in Greater Accra and 16.8% in the North.

In national-level bivariate analyses, social indicators significantly related to the rest variable were age, education, wealth index, Christian religion and literacy (Table 1). In Greater Accra, the significant correlates were age, wealth index, having household electricity, and possession of a refrigerator, a modern cooking stove and a mobile phone (Table 2). In the North, the significant correlates were education, occupation, drinking water source, possession of a motorcycle/scooter, Christian religion, literacy, and having a wall clock and a cupboard (Table 3).

**Table 1:** Rest variable by all social variables, national sample

Putative social determinant indicators	Rest/naps/sleep (%)		Number(weighted)	χ^2^
	**7 or more hours**	**6 or less hours**		

**Age group**				p < 0.000
15-19	91.2	8.8	1,014	
20-24	90.7	9.3	873	
25-29	87.5	12.5	811	
30-34	83.0	17.0	636	
35-39	83.4	16.6	634	
40-44	84.2	15.8	463	
45-49	80.4	19.6	423	
Total	86.8	13.2	4,854	

**Education**				p < 0.000
Secondary or higher	88.4	11.6	2,870	
Primary	87.4	12.6	982	
No education	81.8	18.2	997	

**Occupation**				p < 0.823
White collar	86.9	13.1	3,689	
Agriculture, labour	86.6	13.4	1,116	

**Wealth index**				p < 0.008
Richest quintile	84.8	15.2	1,133	
Richer quintile	86.7	13.3	1,111	
Middle quintile	87.5	12.5	974	
Poorer quintile	90.0	10.0	884	
Poorest quintile	85.4	14.6	752	

**Christian**				p < 0.000
No	80.4	19.6	1,064	
Yes	88.6	11.4	3,788	

**Literacy**				p < 0.001
Can read	88.5	11.5	2,469	
Cannot read	85.2	14.8	2,360	

**Table 2:** Rest variable by social variables, Greater Accra sample

Correlates	Rest/naps/sleep (%)		Number (weighted)	χ^2^
	**7 or more hours**	**6 or less hours**		

**Age group**				p < 0.03
15-19	90.1	9.9	162	
20-24	89.6	10.4	173	
25-29	86.3	13.7	146	
30-34	79.3	20.7	135	
35-39	78.8	21.2	104	
40-44	87.0	13.0	77	
45-49	80.8	19.2	52	
Total	85.4	14.6	849	

**Education**				p < 0.25
Secondary or higher	84.7	15.3	661	
Primary	90.4	9.6	125	
No education	84.4	15.6	64	

**Occupation**				p < 0.53
White collar	85.6	14.4	824	
Agriculture, labour	90.5	9.5	21	

**Wealth index**	86.5	13.5	170	p < 0.000
Richest quintile	73.5	26.5	170	
Richer quintile	88.2	11.8	170	
Middle quintile	89.9	10.1	168	
Poorer quintile	89.5	10.5	172	
Poorest quintile				

**Electricity**				p < 0.02
Yes	84.1	15.9	718	
No	92.7	7.3	123	

**Refrigerator**				p < 0.01
Yes	82.8	17.2	505	
No	89.5	10.5	333	

**Cooking fuel**				
Elec., gas,	85.0	15.0	811	p < 0.03
kerosene	100.0	0.00	27	
Wood, bush, grass				

**Mobile phone**				p < 0.000
Yes	84.2	15.8	759	
No	97.5	2.5	81	

**Table 3:** Rest variable by all social variables, North sample

Correlates	Rest/naps/sleep (%)		Number (weighted)	χ^2^
	**7 or more hours**	**6 or less hours**		

**Age group**				p < 0.27
15-19	88.0	12.0	117	
20-24	86.3	13.7	73	
25-29	77.3	22.7	75	
30-34	78.0	22.0	59	
35-39	78.9	21.1	76	
40-44	88.7	11.3	53	
45-49	83.0	17.0	53	
Total	83.2	16.8	506	

**Education**				p < 0.001
Secondary or higher	94.7	5.3	75	
Primary	89.9	10.2	98	
No education	78.8	21.2	330	

**Occupation**				p < 0.02
White Collar	88.0	12.0	208	
Agriculture, labour	79.8	20.2	292	

**Wealth index**				p < 0.39
Richest quintile	83.8	16.2	99	
Richer quintile	77.6	22.4	98	
Middle quintile	87.6	12.4	105	
Poorer quintile	81.4	18.6	97	
Poorest quintile	84.9	15.1	106	

**Drinking water source**				
Protected	86.5	13.5	421	p < 0.000
Unprotected	67.1	32.9	82	

**Motorcycle/scooter**				
Yes	73.3	26.7	75	p < 0.02
No	84.8	15.2	428	

**Christian**				
No	76.4	23.6	288	p < 0.000
Yes	92.1	7.9	216	

**Literacy**				
Can read	94.8	5.2	96	p < 0.001
Cannot read	80.6	19.4	407	

**Wall clock**				
Yes	73.5	26.5	98	p < 0.004
No	85.6	14.4	404	

**Cupboard**				p < 0.000
Yes	63.2	36.8	76	
No	86.9	13.1	427	

Intercorrelations for all study variables were examined and the data were examined for possible multicollinearity, but no evidence for that was found. In a binary logistic regression analysis using the national data and controlling for age, the significant (p < 0.05) odds ratios were 1.40 for no education compared to secondary and higher education, 0.78-0.43 for the four poorer wealth index quintiles compared to the richest quintile, and 0.55 for Christian religion compared to all others (Table 4). In the data from Greater Accra and controlling for age, the significant (p < 0.05) odds ratios were 2.15 for the second richest wealth quintile compared to the richest quintile and 0.16 for possession of a mobile phone (Table 5). In the data from the North, the significant (p < 0.05) odds ratios were 1.87 for having a protected compared to an unprotected water source, 0.49 for Christian religion compared to all others, and 0.41 for having a cupboard in the home (Table 6).

**Table 4:** Odd Ratios (OR) and 95% Confidence intervals (CI) for reporting rest deprivation amongst Ghanaian women, national sample

Selected predictors	OR	95% CI
**Age group**		
15-19 (ref.)	1	0.72-1.38
20-24	0.99	1.03-1-92
25-29	1.41*	1.37-2.58
30-34	1.88**	1.39-2.62
35-39	1.91**	1.29-2.58
40-44	1.83**	1.64-3.24
45-49	2.31**	

**Education**		
Secondary & higher (ref)	1	
Primary	1.11	0.84-1.45
No Education	1.40*	1.03-1.89

**Wealth index**		
Richest quintile (ref.)	1	
Richer quintile	0.78*	0.61-1.00
Middle quintile	0.71*	0.54-0.92
Poorer quintile	0.52**	0.39-0.80
Poorest quintile	0.43*	0.43-0.80

**Christian**		
No (ref.)	1	0.45-0.67
Yes	0.55**	

**Literacy**		
Can read (ref.)	1	0.78-1.29
Cannot read	1.00	

**-2 Log likelihood**	3,625.03	

**Cox & Snell R Square**	0.026	

Note * Significant at p < 0.05,

** p < 0.001, ref = reference category

**Table 5:** Odd Ratios (OR) and 95% Confidence intervals (CI) for reporting rest deprivation, Greater Accra sample

Selected predictors	OR	95% CI
**Age group**		
15-19 (ref.)	1	0.47-2.04
20-24	0.98	0.66-2.74
25-29	1.34	1.01-4.03
30-34	2.02*	1.24-5.12
35-39	2.54*	0.49-2.74
40-44	1.16	0.95-5.52
45-49	2.28	

**Wealth index**		
Richest quintile (ref.)	1	
Richer quintile	2.15*	1.20-3.84
Middle quintile	0.88	0.45-1.72
Poorer quintile	0.88	0.40-1.95
Poorest quintile	1.65	0.66-4.13

**Household has electricity**		
Yes (ref.)	1	0.31-1.56
No	0.70	

**Household has refrigerator**		
Yes (ref.)	1	0.44-1.54
No	0.83	

**Cooking fuel**		
Elec., gas, kerosene (ref.).	1	--
Wood, bush, grass, etc.	0.00	

**Household has mobile phone**		
Yes (ref.)	1	0.04-0.72
No	0.16*	

**-2 Log likelihood**	643.43	

**R Square, Cox & Snell/Nagelkirke**	0.06/0.11	

Note * Significant at p < 0.05, ref = reference category

**Table 6:** Odd Ratios (OR) and 95% Confidence intervals (CI) for reporting rest deprivation, North sample

Selected predictors	OR	95% CI
**Education**		
Secondary or higher (ref.)	1	0.23-8.73
Primary	1.43	0.28-13.37
No education	1.95	

**Occupation**		
White collar (ref.)	1	0.70-2.26
Agriculture, labour	1.26	

**Water source**		
Protected (ref.)	1	1.04-3.38
Unprotected	1.87*	

**Household has motorcycle/scooter**		
Yes (ref.)	1	0.37-1.32
No	0.69	

**Christian**		
No (ref)	1	0.26-0.92
Yes	0.49*	

**Literacy**		
Can read (ref.)	1	0.34-10.37
Cannot read	1.86	

**Household has wall clock**		
Yes (ref.)	1	0.30-1.04
No	0.56	

**Household has cupboard**		
Yes (ref.)	1	0.22-0.77
No	0.41*	

**-2 Log likelihood**	391.51	

**R Square, Cox & Snell/Nagelkerke**	0.10/0.18	

Note * Significant at p < 0.05, ref = reference category

## Discussion

This study examined national and urban/rural prevalence's of rest deprivation in samples of Ghanaian women, and the association of social indicators of living conditions with rest deprivation. Regarding the three prevalence estimates, ranging from 13% nationally to 16.8% in the North, the only somewhat comparable findings are from the USA's 2006 BRSS [21], in which the prevalence of rest deprivation was 25%. However the exact measures used were different in the USA and the Ghana studies, and the Ghana measure suffers from not have a defined period, such that the period prevalence in not known. The BRSS measure has a period of the past 14 days. In the Ghana data collection interview, the question immediately prior to the rest question was about physical activity and had a response frame of the last seven days. If this response frame remained salient when the rest question was posed, then the period of the prevalence estimate is one week, half that of the period in the BRSS study. However, this is merely conjecture, and illustrates the need for survey research to be very clear about the reporting period when collecting data on past behaviour/experience. Research is also needed on the validity of self-report measures of rest pattern, and it is a limitation of this study that the validity of the rest measure has not been ascertained.

In the regression analysis with the national data, age was a risk indicator at age category 25-29 (and all the older categories), compared to age category 15-19. In the Greater Accra data, age was a risk indicator at age 30-34 and higher, compared to the age category 15-19. In the North analysis, age was not a risk indicator. This is not due to differences in the distributions of age; the largest age distribution difference was 5.3 percent for 35-39 years olds. We conclude that for unknown reasons, the rest variable did not vary significantly with age in the North, while it did vary significantly with age in the national sample and in Greater Accra. This is evidence for a position that generalisation from national contexts to regional contexts should only be done when there is an empirical basis to do so.

Similarly, education was significantly associated with the rest variable in national data but not in regional data. Unlike age, there was a large difference in the distribution of education categories by region; the proportion reporting secondary or higher education versus no education was 59% versus 21% in the national sample, 78% versus 8% in Greater Accra and 15% versus 65% in the North. Thus, the two regions exhibit great heterogeneity on this indicator of living conditions and this in itself is a caution against generalisation of findings from one region to another, or of national findings to regions.

The Wealth Index was significantly related to the rest variable in the national sample, but not in the two regional samples. As for education, there were very large differences in the distribution of the Wealth Index by region. In Greater Accra, using Wealth Index scores generated with the national data, 63.8% were in the richest quintile compared to 6.7 percent in the North. In Greater Accra, 0.3% was in the poorest quintile compared to 54.7% in the North. In Greater Accra, also using national Wealth Index scores, the two richest quintiles accounted for 90.5% of all respondents. In the North, the two poorest quintiles accounted for 72.1% of all respondents. This 'truncation' of the range of wealth is noteworthy, but national wealth scores were not used in this study. Rather, the Wealth Index was recalculated separately for the Greater Accra and the North samples, such that the quintiles were of approximately equal size in all three analyses, about 20% each. Still, one might argue that the concentrations of relative wealth in Greater Accra and poverty in the North reduce variability to such an extent that wealth is not sufficiently variable to be considered as a living conditions indicator. We agree about this possibility, but entertain also another possibility. There is debate in the literature on the social determinants of health about the significance of absolute versus relative levels of living condition indicators. Some argue that absolute levels are of greatest relevance because absolute poverty under certain levels produces conditions for poor health [28]. Others argue that relative levels must also be relevant, since the social gradient in health is evident all along the income spectrum [29]. Illustrating this with the absurd, a man well enough off to own a single engine airplane may be sick with envy because all the neighbours own two engine planes. This is an argument that social position, perhaps alongside wealth, is a determinant of health, and perhaps even more so in very poor societies where education, paid occupation and wealth are relatively scarce. Further, it may be that the social indicators of social position are different in relatively wealthy compared to relatively poor places, such that education, occupation and wealth are most relevant in wealthier places, but not so relevant in poorer places.

This study certainly presents evidence in line with the above reasoning. These exploratory findings suggest that national/international findings from research on the social determinants of health should be generalised to regions only with great caution, if at all. Moving to the heart of the issue, health is variable in poor places as well as wealthy places, and in urban and well as rural areas, as the rest variable data in this paper illustrates. Health in any place is not distributed randomly. Therefore, there exist determinants of health, amongst which there will likely be social determinants. For research on the social determinants of health in very poor regions, a main task is to search for those social determinants, when the classical social determinants are observed to have little explanatory power. Poor regions may be urban or rural, so these issues are equally relevant in both types of places. The result of the above line of reasoning is a call for qualitative, exploratory research in very poor regions in the Global South, to illuminate how living conditions, social position, culture, and health and welfare infrastructure/services affects health.

Moving to other issues, the finding that belonging to a Christian faith (in the national sample and the North sample) is protective against rest deprivation deserves some comment. Belonging to a Christian faith has previously been found to be related to improved health outcomes in Ghana. Women who reported being Christian had higher knowledge of AIDS than women reporting being Moslem or Traditionalist [30]. Also in Ghana, being Christian as opposed to Moslem or Traditionalist was found to predict maternal health service utilization after controlling for the socio-economic variables education and household wealth [31]. The patriarchal culture of Ghana, especially in the North, leaves women with heavy workloads combining livelihood generation and domestic chores with giving birth to and taking care of children, a burden which has been reported to influence several health aspects including sleep [32,33]. Christian culture might moderate the degree of patriarchy in the northern rural areas, thereby to some degree reducing the strain of everyday living for women. In qualitative research by the second author in northern Ghana [34], support for such an effect was observed. Christian culture led to an increased support from husbands to their wives, practically as well as emotionally. The combined effect of religion-related cultural differences might be a reduction of the total life strain for Christian women, leading to increased time for rest. Data on religious affiliations other than the Christian faiths were available, including affiliation with Moslem and traditional faiths. In preliminary analyses, affiliation with either of these was a risk indicator for rest deprivation. This was not pursued as a main point in this paper due the complexity of understanding how cultural indicators such as religious faith are factors in health studies. We wished to avoid the possibility of stigmatisation and chose instead to focus on the protective relationship that Christian faith has with rest deprivation.

The usual measure of wealth is the one used in this study, the Wealth Index. However we also examined possible 'sentinel' possessions, and found that possession of a mobile phone is protective in Greater Accra but not in the North, with the opposite being true for ownership of a cupboard in the North. Individual possessions were included in the analyses in this study in a search for 'sentinel' indicators of social position, that could be used in future survey research if reliably associated with health measures. In this study, neither of these candidate sentinel possessions was reliably associated with rest in multivariate analyses, even if they (and many other candidate sentinel possessions) were significant in bivariate analyses. We conclude that the idea of sentinel wealth indicators may be worth following, but not as a high priority.

Regarding rest deprivation/sufficiency, we know that in Ghana, rest deprivation is highly prevalent, and we may assume that it has significance for health, not from present findings but from the large literature showing that adequate rest and sleep are vital to good health. Further research on rest deprivation is therefore called for; it is to be hoped that the lead of the 2006 BRSS and the 2008 GDHS will be followed by others doing health survey research in the Global South. However, consensus is urgently needed on standardised measurement and reporting periods for prevalence estimates. It may be most prudent to use the 2006 BRSS measure, for which substantial US data are available, and whose two-week period prevalence seems to be a reasonable recall period.

The differing sample sizes are problematic. Power increases with sample size, making it easier to achieve significance in larger than smaller samples, even for effects of similar size. This is well illustrated in the analyses on the predictive power of education on sleep deprivation in the largest and smallest samples (Tables 4 and 6): an OR of 1.40 reaches significances in the national sample, while an OR of 1.95 fails to reach significance in the North. It is reasonable to conclude that this is due to sample size differences. The results from this study should therefore be interpreted with care, especially for analyses comparing national and regional levels. Comparisons between the two regional samples (Greater Accra versus North), can be done with a higher degree of confidence, as the sample sizes are far more similar. In the bivariate analyses for these two regions, no variables emerged as being significant in both samples. This supports the conclusion that caution should be taken when generalizing from findings in one region to another region within a country.

The study is cross-sectional and all the usual caveats about cross-sectional data apply. More importantly, the rest variable is crude, and many important aspects of rest are unmeasured, not least, quality of rest, and proportions of rest from relaxation, naps and sleep. It is laudatory that the 2008 GDHS included an item on rest, but one could have hoped for a few more items as well. Regarding the failure to find an expected relationship between occupation and rest, this may be due to a too crude dichotomisation of the large number of occupations that were classified as white collar or agriculture and labour. Accordingly, we plan to study the associations of occupation and rest in detail, to determine if there is a more sensible way to classify occupation in studies of rest sufficiency. Regarding education, it is quite likely not sufficient to measure only formal education in places were many people have none, as in Northern Ghana. Education for life skills is informal for many people in the world, passed down from generation to generation within families, from elders to the youth in villages, and from artisans to apprentices in communities everywhere. Further work is needed to develop measures for survey research that are sensitive enough to measure levels of informal education, to measure life skills, and to measure social position with validity and reliability.

## Conclusions

Education, occupation and wealth are not related to patterns of rest in two regions of Ghana, even if education and wealth are related to rest patterns in national level data. This study did not illuminate social determinants of rest patterns at regional level, while it did observe that findings at the national level do not generalise to the regional level. Caution is advised in generalising from national data to regions as far as rest patterns are concerned. It is open to further investigation to determine if restricted generalisability is an issue in other countries and for other health endpoints.

We conclude with a point of substantial importance; the social indicators that were examined in this study accounted for very little variance in the rest variable, which is clear from the model fit data in Tables 4, 5 and 6. As mentioned already, we assume that rest patterns do have social determinants, but none of substantial significance was evident among the social variables available in this study. We are convinced that qualitative research in local contexts is needed in order to illuminate the social determinants of rest pattern, and to provide guidance about better ways to measure such determinants in future survey research.

## Competing interests

The authors declare that no competing interests exist.

## Authors' contributions

TB conceived of the study, MBM did the final statistical analyses and wrote the first draft of this paper. MB and TB did preliminary analyses. MBM and TB interpreted the results and contributed to later drafts. Both authors read and approved the final manuscript.

## Pre-publication history

The pre-publication history for this paper can be accessed here:

http://www.biomedcentral.com/1471-2458/10/580/prepub
